# Combination of snapshot hyperspectral retinal imaging and optical coherence tomography to identify Alzheimer’s disease patients

**DOI:** 10.1186/s13195-020-00715-1

**Published:** 2020-11-10

**Authors:** Sophie Lemmens, Toon Van Craenendonck, Jan Van Eijgen, Lies De Groef, Rose Bruffaerts, Danilo Andrade de Jesus, Wouter Charle, Murali Jayapala, Gordana Sunaric-Mégevand, Arnout Standaert, Jan Theunis, Karel Van Keer, Mathieu Vandenbulcke, Lieve Moons, Rik Vandenberghe, Patrick De Boever, Ingeborg Stalmans

**Affiliations:** 1 Department of Ophthalmology, University Hospitals UZ Leuven, Herestraat 49, 3000 Leuven, Belgium; 2 Department of Neurosciences, Research Group Ophthalmology, KU Leuven, Biomedical Sciences Group, Herestraat 49, 3000 Leuven, Belgium; 3 VITO (Flemish Institute for Technological Research), Health Unit, Boeretang 200, 2400 Mol, Belgium; 4 Neural Circuit Development and Regeneration Research Group, Department of Biology, KU Leuven, Naamsestraat 61, 3000 Leuven, Belgium; 5 Laboratory for Cognitive Neurology, Department of Neurosciences, KU Leuven, Herestraat 49, 3000 Leuven, Belgium; 6 Department of Neurology, University Hospitals UZ Leuven, Herestraat 49, 3000 Leuven, Belgium; 7 Imec, Kapeldreef 75, 3001 Leuven, Belgium; 8 Clinical Research Center, Mémorial A. de Rothschild, 22 Chemin Beau Soleil, 1208 Geneva, Switzerland; 9 Division of Psychiatry, University Hospitals Leuven, Herestraat 49, 3000 Leuven, Belgium; 10 Alzheimer Research Center KU Leuven, Leuven Brain Institute, Herestraat 49, 3000 Leuven, Belgium; 11 Hasselt University, Center of Environmental Sciences, Agoralaan, 3590 Diepenbeek, Belgium; 12 Department of Biology, University of Antwerp, Universiteitsplein 1, 2610 Wilrijk, Belgium

**Keywords:** Retina, Brain, Neurodegeneration, Cognitive impairment, Alzheimer’s disease, Amyloid-beta (Aβ), Hyperspectral imaging, Machine learning, Biomarker

## Abstract

**Introduction:**

The eye offers potential for the diagnosis of Alzheimer’s disease (AD) with retinal imaging techniques being explored to quantify amyloid accumulation and aspects of neurodegeneration. To assess these changes, this proof-of-concept study combined hyperspectral imaging and optical coherence tomography to build a classification model to differentiate between AD patients and controls.

**Methods:**

In a memory clinic setting, patients with a diagnosis of clinically probable AD (*n* = 10) or biomarker-proven AD (*n* = 7) and controls (*n* = 22) underwent non-invasive retinal imaging with an easy-to-use hyperspectral snapshot camera that collects information from 16 spectral bands (460–620 nm, 10-nm bandwidth) in one capture. The individuals were also imaged using optical coherence tomography for assessing retinal nerve fiber layer thickness (RNFL). Dedicated image preprocessing analysis was followed by machine learning to discriminate between both groups.

**Results:**

Hyperspectral data and retinal nerve fiber layer thickness data were used in a linear discriminant classification model to discriminate between AD patients and controls. Nested leave-one-out cross-validation resulted in a fair accuracy, providing an area under the receiver operating characteristic curve of 0.74 (95% confidence interval [0.60–0.89]). Inner loop results showed that the inclusion of the RNFL features resulted in an improvement of the area under the receiver operating characteristic curve: for the most informative region assessed, the average area under the receiver operating characteristic curve was 0.70 (95% confidence interval [0.55, 0.86]) and 0.79 (95% confidence interval [0.65, 0.93]), respectively. The robust statistics used in this study reduces the risk of overfitting and partly compensates for the limited sample size.

**Conclusions:**

This study in a memory-clinic-based cohort supports the potential of hyperspectral imaging and suggests an added value of combining retinal imaging modalities. Standardization and longitudinal data on fully amyloid-phenotyped cohorts are required to elucidate the relationship between retinal structure and cognitive function and to evaluate the robustness of the classification model.

## Background

Diagnosing Alzheimer’s disease (AD) is a challenging task. In recent years, the “ATN” categorization, which is a framework for defining AD based on biomarker proxies of pathology, where A stands for amyloid-beta (Aβ) status, “T” for tau, and “N” for neurodegeneration biomarkers, has gained attention [1, 2]. The highest diagnostic accuracy can likely be achieved by combining several ATN biomarkers [3]. However, given the associated cost, invasiveness, and/or potential side effects, amyloid-PET and cerebrospinal fluid (CSF) biomarker analyses are not recommended for screening [1, 4, 5]. These limitations warrant the identification of biomarkers using affordable and non-invasive diagnostic tools [6].

Because of a shared ontogenesis, the retina displays similarities to the brain and spinal cord in terms of anatomy, functionality, response to insult, and immunology. Hence, the eye provides a unique window to the central nervous system without the need for expensive, invasive, and/or potentially harmful examinations [7–9]. One line of investigation is focused on retinal changes occurring in patients with AD. There is increasing evidence pointing to neuroretinal thinning and ganglion cell degeneration, abnormal electrical responses, reduced retinal perfusion, and microvascular changes, as well as elevated retinal levels of Aβ_40_/Aβ_42_ peptides and pTau [10–13]. Retinal nerve fiber layer (RNFL) and macular thinning and loss of the melanopsin-immunopositive subtype of ganglion cells have been documented in early AD patients. Although research on the identification of pathological Aβ accumulation in the human retina is limited and inconsistent, retinal Aβ accumulation and retinal Aβ plaques were detected before their cerebral counterparts in both in vivo and ex vivo transgenic mouse models [13–18]. Collectively, these findings suggest that the retina holds potential to play a major role in early diagnosis of AD, as also suggested by Alber et al. [9].

Optical coherence tomography (OCT) is a non-invasive, high-resolution diagnostic tool capable of generating cross-sectional coupes of the retina and choroid. Studies using this imaging modality have demonstrated the thinning of the RNFL, mostly in the superior and inferior quadrants, and the macular ganglion cell complex [19]. This reflects the loss of the retinal ganglion cell complex and thereby corroborates the findings from postmortem histological studies [13, 20–25]. Although RNFL and macular ganglion cell complex thickness have been inversely correlated with disease duration and severity [26–28], longitudinal data to support the significance of OCT imaging are not available, and the diagnostic accuracy of RNFL changes alone is probably insufficient due to its low specificity. The newer generation of spectral domain OCT devices offers a markedly improved signal-to-noise ratio. Nonetheless, imaging the elderly poses an additional challenge due to the possibility of media opacities such as cataracts [29] and an impaired ability to focus properly. Most commercially available OCT devices offer an image quality indicator to assess scan quality.

Building on the unique biochemical properties of Aβ, different imaging techniques have been developed with the aim to detect changes caused by the presence of retinal Aβ in vivo. One such imaging technique is based on the use of the fluorochrome curcumin that binds to Aβ. This approach has shown promising results, with a retinal Aβ index that correlates well with cerebral Aβ plaques [14, 30–32]. Another such imaging modality, hyperspectral retinal imaging (HSRI), which was recently reviewed [33], is a label-free imaging technique. This technique allows one to quantify a decrease in the spectral reflectance of retinal and cerebral tissue of AD subjects at wavelengths between 460 and 570 nm. This spectrum may be indicative of increased Rayleigh scattering due to the presence of Aβ [34]. Postmortem studies in both animal and human retinas, and in vivo studies in rodents, have shown that HSRI can detect spectral changes that could potentially be caused by the presence of retinal Aβ aggregates provided that there are such aggregates in the human retina [34, 35]. It has to be noted, however, that HSRI does not directly visualize retinal Aβ deposition, but records a spectral shift that could be explained by the presence of retinal protein deposits in certain stages of aggregation, given the relationship between particle size and different types of light scattering. We cannot exclude that factors other than amyloid deposition may underly a spectral shift in AD versus controls. It has recently been shown that machine learning methods using HSRI data are capable of distinguishing between amyloid-PET-positive cases and controls in a clinical setting [36], which is in line with the assumption that these spectral differences are due to the retinal accumulation of Aβ in amyloid-PET-positive cases. However, it has to be born in mind that the pathological correlates of these recently reported spectral changes in AD patients’ retinas have not yet been identified, and as such, alternative explanations (e.g., tau accumulation, neuro-inflammation) cannot be excluded.

This proof-of-concept clinical study investigates whether bimodal retinal image analysis, using both HSRI and OCT, can differentiate between AD patients (cases) and controls. The current study combines two elements of the “ATN” categorization framework: a snapshot HSRI setup for in vivo detection of a spectral retinal shift presumably related to Aβ presence (“A”) is deployed, and the neurodegeneration pillar (“N”) is assessed by quantifying changes in RNFL thickness using OCT.

This study examined the diagnostic performance of a set of ophthalmological measures in a clinical cohort. In such a cohort, patients who have received a prior diagnosis based on standardized clinical diagnostic criteria are consecutively recruited. Technology assessment in a clinical cohort may be hampered by potentially lower diagnostic accuracy compared to a research cohort. However, this disadvantage is at least partly counterbalanced by the fact that a clinical cohort may be more representative for the population where this novel technology will be implemented.

## Materials and methods

### Study design

This single-center cross-sectional academic memory-clinic-based study was executed during June to September 2019 at University Hospitals UZ Leuven, Department of Ophthalmology (Leuven, Belgium). An overview of the study with the most important steps in data analysis is provided in Additional file 1: Fig. A1. The study adhered to the principles of the European Union Directive on Clinical Trials (2001/20/EC) and all requirements for the provisions of the Declaration of Helsinki (World Medical Association, Edinburgh, 2000). Approval was issued by the Ethics Committee of the University Hospitals Leuven before the study commenced (reference number S59048).

### Participant recruitment

Participants were consecutively recruited from an academic memory-clinic-based cohort. The clinical diagnostic workup in 7 of the 17 participants included either Fujirebio ELISA for Aβ42, total tau, and ^181^phosphotau or EuroImmun ELISA of Aβ42, Aβ40, and total tau in CSF. The cut-offs used were based on Adamczuk et al. [37]. One out of 7 also underwent [^11^C]-Pittsburgh compound B amyloid-PET. In all 7, this led, together with the clinical evaluation, neuropsychological assessment, and imaging investigations, to a diagnosis of biomarker-proven AD in the dementia phase.

In the ten remaining participants, a diagnosis of clinically probable AD according to the National Institute on Aging-Alzheimer’s Association (NIA-AA) criteria [38] was made based on a clinical evaluation by a cognitive neurologist (RV), blood examination, detailed neuropsychological assessment (performed in 8 out of 10) revealing a cognitive profile characteristic of AD, MRI (performed in 9 out of 10) or CT (in one) for the exclusion of cerebrovascular disease that could explain the cognitive decline, and in selected cases [^18^F] fluorodeoxyglucose PET (FDG PET) (performed in 3 out of 10) demonstrating a pattern characteristic of AD. All cases underwent six-monthly neurological visits for several years (ranging from 1 to 10.5 years of follow-up). The disease course following the diagnosis was in agreement with AD in all cases, including gradually progressive cognitive decline with relative preservation of personality as well as the absence of clinical neurological signs beyond the cognitive changes. Ten out of 11 in whom Apolipoprotein E (APOE) status was available were ε4 carriers, and one was ε3 homozygous carrier. All cases were in an early or moderate dementia stage at the time of study inclusion, with AD diagnosis based on thorough clinical workup and follow-up, without amyloid biomarker confirmation.

CSF was collected, stored, and analyzed as described by Adamczuk et al. [37]. Lumbar punctures were carried out at the L4/5 level in the morning (10 a.m.–2 p.m.) and collected in polypropylene tubes (total volume 15 ml, Greiner Bio-one Cellstar; VWR, Leuven, Belgium), discarding 1 ml to avoid traumatic blood contamination. Samples were centrifuged within 30 min after collection (2600 rpm, 10 min, 4 °C). After centrifugation, supernatants were transferred into polypropylene tubes and from there aliquoted in 1.5 ml polypropylene tubes (1 ml volume CSF/tube; Kartell, Noviglio, Italy). In samples collected prior to 2018, the CSF AD biomarker assay used was the Fujirebio ELISA for Aβ42, total tau, and ^181^phosphotau; thereafter, the Euroimmun ELISA for Aβ42, Aβ40, and total tau was used. Tests were performed at the Laboratory Medicine Department of UZ Leuven, Belgium, in a ISO-15189 and Joint Commission International-accredited environment by an expert technician. Cut-offs are based on Adamczuk et al. [37]: Fujirebio: Abeta42 853 pg/ml, total tau 352 pg/ml, phosphotau 86 pg/ml, Abeta42/total tau 2.258; Euroimmun Abeta42 745 pg/ml, Abeta42/Abeta40 0.096, total tau 436 pg/ml, Abeta42/total tau 2.006 [37]. MRI was performed on a 3-T clinical MRI scanner. [^18^F]-FDG PET scans were acquired using a HiRez PET-CT camera (Siemens) operated in 3-dimensional mode. ^18^F-FDG (150 MBq) was injected intravenously under standard conditions, that is, subjects lying supine in a dimly lit, quiet room, with ears and eyes open. Thirty minutes after ^18^F-FDG injection, a dynamic scan of 30 min (6 frames of 5 min each) was started. During the acquisition, the subject’s head was immobilized by means of a vacuum pillow. ^18^F-FDG images were reconstructed using iterative ordered-subset expectation maximization (4 iterations, 4 subsets). Visual readings were based on Z map renderings in line with current guidelines [39]. [^11^C]-Pittsburgh compound B amyloid-PET images were acquired on a TruePoint Siemens PET scanner using static acquisition during an acquisition window of 40–70 min post-injection. A low-dose computed tomography scan was performed for attenuation correction, prior to the PET scan. Results are based on visual reads by an accredited nuclear medicine physician with special expertise in amyloid imaging. Diagnostic information for all 17 AD cases is presented in Table 1.

**Table 1:** Available diagnostic information for all AD cases

AD subject	Age (years)	MMSE	Neuropsychol. assessment	Structural MRI	FDG PET	Amyloid-PET	CSF	Duration of follow-up (years)
1	82	15	+	+/−	−	−	−	10.5
2	69	18	+	−	+	−	−	1
3	63	22	+	+	−	−	−	2
4	67	27	+	+	−	−	−	4.5
5	62	≤ 8*	+	+/−	−	+	+	3.5
6	73	17	+	+	−	−	−	1.5
7	74	10	–	+	+	−	−	2
8	76	14	–	+	−	−	−	1
9	71	15	+	+	+	−	+	7
10	81	17	+	+	−	−	−	6
11	79	22	+	+	+	−	+	1
12	77	20	+	+/−	+/−	−	+	4
13	72	22	+	+	+	−	−	3.5
14	70	14	–	+	+	−	+	1.5
15	73	24	+	+	−	−	−	1.5
16	75	24	+	+	+	−	+	5
17	58	10	–	+	+	−	+	1.5

*MMSE* Mini-Mental State Examination score at the time of testing, *Neuropsychol. assessment* neuropsychological assessment as part of the diagnostic workup, + performed and in accordance with an AD diagnosis, − not done, +/− performed but not contributive. APOE genotypes are not provided for confidentiality reasons

*MMSE no longer possible at the time of ocular imaging; noted score is the latest available one

Non-amyloid-phenotyped controls (Mini-Mental State Examination (MMSE) scores 29–30) were recruited from the family and/or caretakers accompanying the AD patients as well as the Department of Ophthalmology UZ Leuven. Subjects were recruited only if they were able to provide written informed consent. Exclusion criteria included an age of under 55 or above 85 years, a visual acuity worse than 20/40, presence of glaucoma or occludable anterior chamber angle, an insufficient clarity of optical media to allow retinal imaging, a personal medical history of retinal neovascularization or retinal dystrophy, or the presence of retinal drusen, as well as the presence of neurological comorbidities. A total of 41 subjects met the above criteria (18 AD, 23 controls). One AD subject and one control subject were excluded from further analysis due to the poor quality of their hyperspectral images.

### Patient examination and imaging procedures

#### General history and general ocular examination

Subjects filled in a questionnaire about their general and ocular health history. Visual acuity (VA (logMAR)) was determined in both eyes, and the better eye was included for further examination and imaging. In case of symmetric visual acuity, one eye was randomly chosen. A general ophthalmological examination of the eye being studied was performed, including biomicroscopy, keratometry, and intraocular pressure (IOP) measurement by pneumotonometry using Tonoref II (Nidek Co Ltd., Aichi, Japan); dilated fundoscopy (tropicamide 0.5% and phenylephrine 2.5%), stereoscopic optic disc photography, and macula-centered fundus photography using the Visucam PRO NM (Carl Zeiss Meditec AG, Jena, Germany); and ultra-widefield scanning laser ophthalmoscopy (UWF-SLO) imaging using Optomap Daytona P200C UWF-SLO (Optos Plc, Dunfermline, UK).

#### Hyperspectral retinal imaging

HSRI was performed with a XIMEA SNm4x4 VIS hyperspectral snapshot camera (Ximea, Münster, Germany; XIMEA CamTool software version 4.11) connected with a C-mount to a TL-230T relay lens (Topcon Corporation, Japan), installed on a Topcon TRC-50DX fundus camera (Topcon Corporation, Japan) (see Additional file 1: Fig. A1).

The XIMEA snapshot camera contains a hyperspectral sensor from IMEC. This mosaic pattern of pixel-size spectral filters is integrated on top of a standard complementary metal oxide semiconductor (CMOS) sensor (1088 × 2048 pixels). This allows acquiring spatial and spectral information (460–620 nm, 10-nm bandwidth) in one capture (272 × 512 pixels) without the need for wavelength or spatial scanning by combining 4 × 4 imaging pixels into hyperspectral pixels with 16 spectral bands [40, 41]. Settings for the image acquisition consisted of an exposure time of 0.2 ms, 50-degree field of view, and no background illumination. Macula-centered images were recorded. The first image of each study eye was captured with a flash intensity of 50 Ws, which was subsequently increased or decreased to capture an image with maximum light intensity while avoiding saturation outside the optic nerve head (ONH). For the patient, the acquisition of one hyperspectral snapshot image implies exposure to one flash of low to moderate intensity during an acquisition time of 0.2 ms, while focusing on an external fixation light.

#### Optical coherence tomography

OCT measurements were performed and analyzed using the RT-vue XR Avanti (Optovue, Fremont, CA, USA; software version 2015.1.1.98). RNFL thickness and vertical cup-to-disc ratio (vCDR) were recorded from the ONH report. RNFL thickness was measured over 360° (RNFL_AVG_, average) and per quadrant (RNFL_SUP_, superior; RNFL_NAS_, nasal; RNFL_INF_, inferior; RNFL_TEM_, temporal).

### Basic statistics

Statistical analyses on patient characteristics and OCT parameters were performed using SPSS 26.0 for Windows (SPSS Inc., Chicago, USA). After evaluation of the distribution of the results for normality, differences were analyzed using an independent sample *t* test for continuous parameters. Ordinal and dichotomous parameters were compared using the Mann-Whitney and chi-square testing, respectively. Analysis of RNFL parameters has been corrected for age, gender, and scan quality using multivariate linear regression. Statistical significance was determined based on two-sided *P* values of < 0.05. A Bonferroni correction for multiple comparisons has been applied to the RNFL parameters.

### Hyperspectral image analysis

#### Definition of regions of interest

**Fig. 1:**
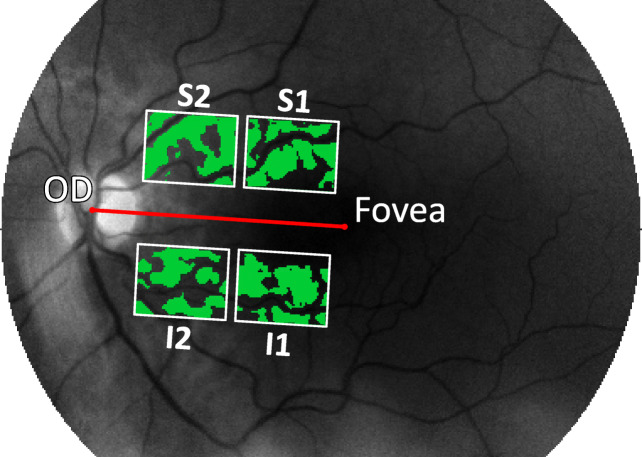
Illustration of the positioning of the 4 rectangular regions of interest. Regions are indicated by superior 1 (S1), superior 2 (S2), inferior 1 (I1), and inferior 2 (I2). The green zones refer to the parts in the image that were used in the analysis after removing the retinal blood vessels. OD refers to the optic disc

Raw reflectances were first converted to relative reflectances using the preprocessing steps outlined in Additional file 2: Preprocessing. Subsequently, pixels corresponding to blood vessels were identified as described in Additional file 2: Removal of retinal blood vessels and discarded for further analysis. Four rectangular regions of interest (ROIs) as determined relative to the line going through the center of the optic disc (OD) and fovea were defined for standardization of analysis between subjects (Fig. 1). This approach is comparable to the one described by Hadoux et al. [36] The a priori selection of ROIs limits the risk of diluting a possibly weak Aβ signal when considering the entire retina and of detecting random effects when considering a greater number of regions. In this study, locations of the center of the OD and the fovea were marked manually. Each of the ROIs has a height of 40 pixels and a width equal to 35% of the distance between the center of the OD and the fovea. The width of the ROIs was defined relative to the OD-fovea distance to guarantee that the ROIs did not overlap. The range of the widths of the ROIs varied between 32 and 52 pixels. The relative reflectance values of the spectrum were averaged in the four individual ROIs and standardized using the procedure described in Additional file 2: Standardization, resulting in a 14 × 1 vector for each ROI.

### Classification model’s training and evaluation

Linear discriminant analysis (LDA) classifiers were trained to distinguish AD subjects from controls. LDA was chosen as it is a linear classifier that does not require hyperparameter tuning, which makes it suitable for our use case as we do not have a large enough sample size to train more complex models and do associated hyperparameter tuning. Models were trained using scikit-learn library (version 0.21.3) in a Python programming language environment [42]. In the model selection procedure, performances obtained for the predefined ROIs and their combination with RNFL features were compared. ROIs were included in the model selection procedure if the standard error of the mean (SEM) intervals of the average spectrum for AD patients and controls did not overlap for at least one wavelength. For each ROI, two input configurations for the classifier were considered: one that consisted of normalized hyperspectral data, and one that combined normalized hyperspectral data and 5 RNFL features, one for each OCT quadrant and the averaged value over the 4 OCT quadrants. The performance of the selected ROIs, with and without combining the spectra with RNFL features, was compared using nested leave-one-out cross-validation (LOOCV). Note that we limit the number of regions that we consider and the associated feature combinations (i.e., only consider spectra with or without RNFL features, without doing further extensive feature selection) with the aim of reducing the risk of overfitting in the model selection procedure. We refer to Additional file 2: Nested leave-one-out cross-validation for a brief description of nested LOOCV and to Varma et al. [43] for a more detailed description.

## Results

### Patient characteristics

The cohort consisted of 17 participants with AD and 22 cognitively intact controls, as described under the “Participant recruitment” section. There were no significant differences in age and sex distribution between the AD and the control groups. MMSE, the best corrected visual acuity (BCVA), prevalence of pseudophakia, and RNFL thickness (average and inferior) were statistically significantly lower in the AD group. An overview of demographical and clinical characteristics is given in Table 2.

**Table 2:** Demographical and clinical characteristics

Parameter	Alzheimer’s disease (AD) patients (*n* = 17)	Controls (*n* = 22)	*P*
Time since AD diagnosis (years)	2.7 ± 2.6	NA	–
Age (years)	71.9 ± 6.6	68.6 ± 8.4	0.193*
Sex (male/female)	7/10	12/9	0.267^‡^
Body mass index (kg/m^2^)	24.9 ± 2.9	26.0 ± 4.4	0.412*
Eye (right/left)	10/7	10/12	0.408^‡^
MMSE	17.6 ± 5.5	29.3 ± 0.9	**< 0.001**^†^
BCVA (logMAR)	0.14 ± 0.11	0.06 ± 0.08	0.027*
IOP (mmHg)	14 ± 3	15 ± 4	0.359*
Phakic (yes/no)	15/2	12/10	**0.024**^‡^
Vertical cup/disc ratio	0.52 ± 0.15	0.51 ± 0.21	0.878*
RNFL_AVG_ (μm)	84.8 ± 7.5	92.1 ± 7.3	**0.005**^§^
RNFL_SUP_ (μm)	104.2 ± 8.9	109.8 ± 12.4	0.019^§^
RNFL_INF_ (μm)	104.3 ± 11.1	115.6 ± 11.4	**0.009**^§^
RNFL_TEM_ (μm)	63.3 ± 8.1	70.4 ± 6.7	0.069^§^
RNFL_NAS_ (μm)	66.3 ± 11.7	72.7 ± 8.6	0.012^§^

*MMSE* Mini-Mental State Examination, *BCVA* best corrected visual acuity, *RNFL* retinal nerve fiber layer. Data are presented as mean ± standard deviation

*Independent samples *t* test

^†^Mann-Whitney *U* test

^‡^Chi-square test

^§^Multivariate linear regression corrected for age, gender, and image quality

### Results of multimodal image analysis

**Fig. 2:**
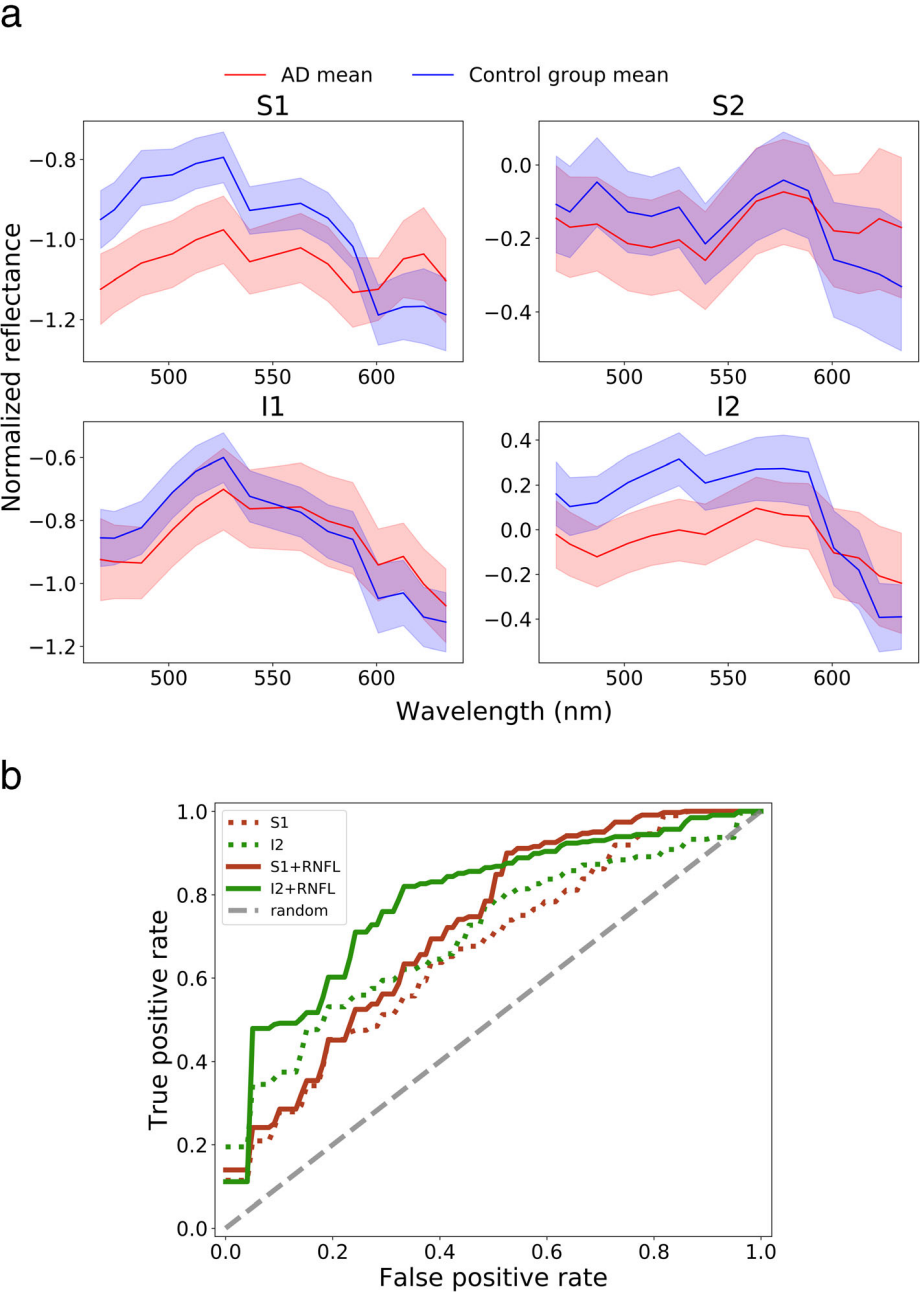
**a** Mean spectra in the 4 ROIs after normalization. Shaded areas indicate the mean ± the standard error of the mean. S1 and S2 refer to the superior regions, and I1 and I2 refer to the inferior regions (cfr. Fig. 2). **b** Average receiving operating characteristic (ROC) curves over all inner loop cross-validation runs for all configurations. S1 and I2 refer to models taking only spectra as input, and S1+RNFL and I2+RNFL refer to models with both spectra and retinal nerve fiber layer (RNFL) thickness as input

Normalized mean reflectance spectra are shown in Fig. 2a. For ROIs, S1, and I2, the SEM values did not overlap between AD and controls for at least one wavelength, indicating that the population mean of AD subjects is different from that of controls. These ROIs were selected for the model selection procedure. Hence, four configurations were considered as input to the machine learning model: S1 spectra, I2 spectra, S1 spectra + RNFL thickness, and I2 spectra + RNFL thickness. The first two configurations consist of 14 input features, one for each wavelength. The last two configurations consist of 19 input features, one for each wavelength and 5 additional ones that represent RNFL thickness values.

The inner LOOCV loop results from the LDA allow comparing the different model configurations. Figure 2b shows the average ROC curves for the inner cross-validation (CV) runs. For the S1 region, the average area under the curve (AUC) is 0.67 (95% CI [0.51, 0.83]) with only spectra as input to the model and 0.72 (95% CI [0.57, 0.88]) with spectra and RNFL features as input. For the I2 region, the average AUC is 0.70 (95% CI [0.55, 0.86]) and 0.79 (95% CI [0.65, 0.93]), respectively. Inclusion of the RNFL features resulted in an improvement of the AUC in both the S1 and I2 regions.

**Fig. 3:**
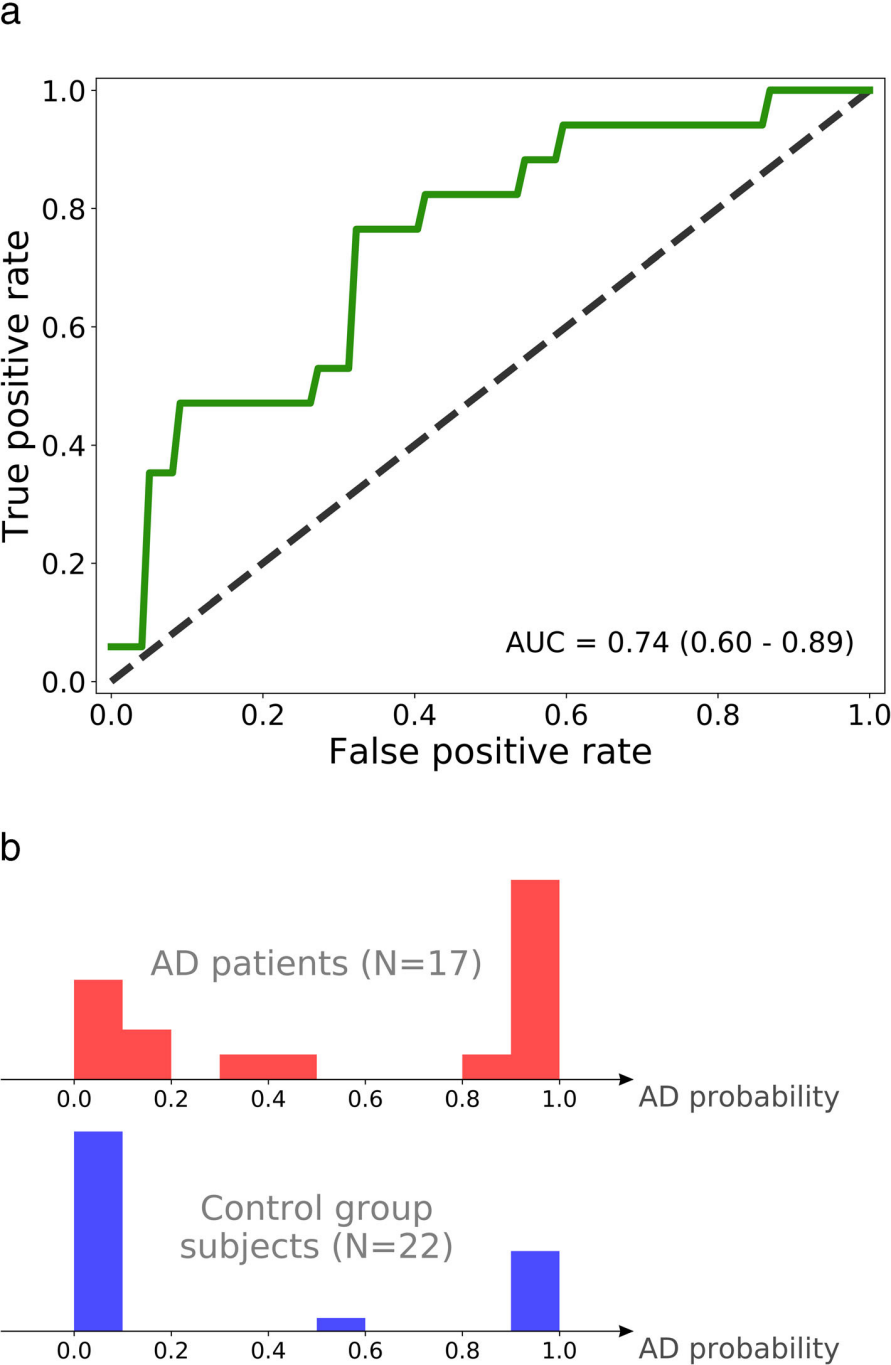
**a** Receiver operating characteristic (ROC) curve generated through nested leave-one-out cross-validation (LOOCV). Area under the curve (AUC) given with 95% confidence interval. **b** Distribution of AD probabilities. Probabilities predicted by the models in the outer LOOCV loop for AD patients (top) and cognitively intact elderly (CIE) subjects (bottom)

The results of the inner LOOCV runs consistently provided the I2 region combined with RNFL thickness values to be selected for validation in the outer loop. The I2 region and RNFL thickness values were selected in 38 out of 39 inner runs. Figure 3a shows the final ROC curve generated for predictions in the outer LOOCV loop. An AUC of 0.74 with a 95% confidence interval of [0.60–0.89] was obtained. The AUC generated in this nested LOOCV is an unbiased estimate according to Varma and Simon [43]. Fifteen out of the 22 controls had a probability of having AD close to 0, and 9 out of 17 AD patients had a score near 1. Figure 3b shows the distribution of the AD probabilities that were produced in the outer LOOCV loop. There were no significant differences in non-retinal parameters between AD patients with high and low probability scores. Of note, comparison of spectral properties between biomarker-proven and non-biomarker-proven AD subjects did not reveal any significant difference.

## Discussion

This clinical study reports a proof-of-concept for a bimodal imaging approach using hyperspectral and OCT imaging to detect retinal changes related to Aβ presence. All retinal data were fed to a dedicated analysis pipeline to discriminate AD patients from controls. The performance of the current machine learning model improved with the addition of peripapillary RNFL data as input, underlining the added value of the bimodal imaging approach. Within the field of glaucoma, the most prevalent ocular neurodegenerative disorder, peripapillary RNFL measurements are the most standardized across the various available OCT devices [44], partly because this is one clearly defined anatomical layer. Thinning of the peripapillary RNFL is directly associated with the structural loss of ganglion cell axons in the retina of glaucoma patients [45]. Significant thinning of the RNFL_AVG_ was observed in AD patients, most pronounced in the inferior quadrant. Previous cross-sectional studies using OCT have demonstrated that RNFL thinning in AD patients is not uniform and most pronounced in the superior and inferior quadrants. Of note, anatomically, the RNFL fibers converge in the superior and inferior quadrants, giving rise to the characteristic “double-hump” pattern of RNFL thickness and rendering those quadrants more discriminatory for changes in thickness [16, 19]. From a meta-analysis by den Haan et al., it has been shown that mean peripapillary RNFL thickness is decreased by 9.70 μm in AD versus control, with a larger effect for time domain OCT compared to spectral domain OCT. [19] Chan et al. performed a meta-analysis limited to studies using spectral domain OCT and reported a decrease in mean peripapillary RNFL of 5.99 μm [46]. These findings are in line with our study, which was performed with spectral domain OCT, and where a decrease in mean peripapillary RNFL of 7.7 μm (*P* = 0.008) was noted. Both longitudinal studies that have been published so far consistently indicate that specifically in the inferior quadrant RNFL thinning is associated with progression of cognitive decline in AD patients [47, 48].

In the current study, an AUC of 0.74 (CI 0.60–0.89) was obtained using a combination of hyperspectral and RNFL data, whereas Hadoux et al. [36] report an AUC of 0.82 (CI 0.67–0.97) on their principal validation set consisting of fellow eyes of training subjects, and an AUC of 0.87 (CI 0.69–1.0) on a separate validation cohort of 4 AD patients. While Hadoux et al. used only hyperspectral data in their machine learning pipeline, the current study reports the training of a multimodal model and a validation using nested LOOCV. But there are also important differences in hardware characteristics: whereas the off-the-shelf available snapshot camera used in the current study relies on a mosaic pattern of pixel-size spectral filters integrated on top of a standard CMOS sensor, Hadoux et al. made use of a wavelength scanning HSRI technique (metabolic hyperspectral retinal camera (MHRC), Optina Diagnostics, Montreal, Canada). The latter outweighs the spectral and spatial resolution of the snapshot camera, at the expense of longer acquisition times and higher hardware cost. This probably accounts for the difference in the AUC estimate between this study and the study by Hadoux et al., but it should be pointed out that both CIs entirely overlap and thus cannot be considered significantly different. Most importantly, the current study shows that with a cheaper and faster HSRI technique, boosted with data from the already widely available OCT technique, comparable results can be achieved. More et al. [35] provide a biostatistical analysis of the differences in optical density between AD subjects and controls, but they did not develop a classification model and consequently did not report performance results that allow for a direct comparison with the present study. Sharafi et al. [49] also trained a classifier to distinguish AD subjects from controls based on hyperspectral images. They extracted vessel tortuosity and diameter as well as several spatial-spectral texture measures in different retinal anatomical regions. Their best model obtained a classification accuracy of 85%. Sharafi et al. [49] used single-level CV to perform both model selection and evaluation, which may have resulted in over-optimistic performance estimates [43]. Of note, in the present study, a nested LOOCV was used to obtain an unbiased estimate of performance. This method is more appropriate for smaller sample sizes and probably reflects the true accuracy better than single-level CV.

In the present study, four ROIs positioned relative to the OD-fovea line were selected to ensure consistent sampling locations. Similar to Hadoux et al. [36], the largest HSRI differences were observed in the S1 region (cfr. Fig. 2a). The subsequent model selection procedure, however, identified the I2 region as the most informative one to discriminate between AD and controls, both when considering HSRI results only and when based on a bimodal approach combining HSRI and OCT. Concerning the spectral shifts measured by HSRI, one could hypothesize that the I2 region shows relative differences compared to the other regions regarding blood flow, retinal vascular reactivity, tissue composition and permeability, and light stimulation, which could make it more susceptible to deposition/less susceptible to clearance of proteins, such as amyloid. Within the field of glaucoma, the most prevalent ocular neurodegenerative disorder, the inferior temporal peripapillary neuronal tissue is most prone to glaucomatous damage resulting in thinning of the retinal neuronal tissue. It has been postulated that differences in vascular hemodynamics (less responsive to vasodilation and more responsive to vasoconstriction) might contribute to this finding [50]. Such differences could equally contribute to regional differences in protein deposition and clearance, affecting HSRI results. The largest difference in relative reflectance spectra in the current study was observed at shorter wavelengths (< 550 nm), which is consistent with the observations by More et al. [35] and Hadoux et al. [36]. While further research is needed to ensure that the observed effects are not due to other factors than the presence of retinal Aβ, More et al. [34] have previously substantiated the hypothesis that the observed spectral effects observed in these wavelengths were caused by the presence of soluble Aβ in the retina. This hypothesis is based on a simulation of the light paths through the different retinal layers and the proposition that the accumulation of soluble Aβ aggregates in the retina causes additional Rayleigh scattering over time, which leads to a reduction in measured light at shorter wavelengths within a recording aperture [41]. The issue of retinal accumulation of Aβ in AD remains controversial, with divergent results across research groups and studies, which might at least partly be explained by the significant heterogeneity in techniques for staining and tissue preparation, and in study design [9]. Opposite each of the handful of studies that support the hypothesis of retinal Aβ in AD [13, 14, 34–36, 51, 52], one can be put that could not confirm the presence of Aβ plaques in the human AD retina using immunohistochemistry [53–56]. Nevertheless, further standardization of ex vivo and in vivo methods is crucial to evolve towards clearance of this controversy. Such studies, investigating pathological processes in the retina, such as protein depositions of Aβ or tau, but also neuro-inflammation, are required to assess what underlies the spectral changes in AD retinas.

### Limitations

The present study should be interpreted within the context of its strengths and limitations. First, the lack of biomarker confirmation in the majority of subjects is a limitation. Nevertheless, although only 7 out of 17 AD subjects had a biomarker-proven AD diagnosis, they all fulfilled the widely used NIA-AA criteria for the diagnosis of probable AD [38]. To assess the diagnostic performance of the ophthalmological markers, the study participants were recruited consecutively from an academic memory-clinic-based cohort of patients who had received a prior diagnosis of either biomarker-proven AD or clinically probable AD in an early or moderate dementia stage. The diagnostic investigations which led to a diagnosis of AD were thorough, but CSF or amyloid-PET biomarker tests were only available for those patients in whom this was considered clinically indicated. Although the value of the current results obtained in a clinical cohort is considerable, future research building on the results of this pilot study should focus on data collection in fully Aβ-phenotyped cohorts. Second, the HSRI setup used here relies on snapshot imaging. This is both a strength and a limitation. Previous studies [35, 42] have used the MHRC, which offers superior spatial and spectral resolution, but requires longer acquisition times. On the other hand, More et al. [34, 35] have developed a custom imaging system that simultaneously captures a conventional two-dimensional retinal image and a spectral image along one dimension. While this setup provides short acquisition times, it only provides spectral information along a single horizontal line. The XIMEA snapshot camera in the current setup overcomes several of these issues. Spatial and spectral information is obtained in one take, thus enabling real-time data acquisition which is crucial to avoid eye movements in retinal imaging, although at the cost of spatial and spectral resolution [40, 41]. Third, the current study included only peripapillary OCT data. Further research into the use of macular OCT parameters, such as the ganglion cell complex and the ganglion cell-inner plexiform layer complex, within the proposed model for bimodal retinal imaging in AD is a path that should be explored in the future. Finally, the robust statistics using the LOOCV approach in this study partly compensate for the limited sample size. The limited sample size was also the motivation to select only the regions for which the SEM did not overlap for at least one wavelength in the model selection procedure. While this selection could introduce a selection bias, it also mitigates the risk of overfitting by reducing the number of configurations to consider.

The results of the current study provide potential for future research. First, these findings should be confirmed in a larger, fully Aβ-phenotyped cohort with an assessment of the association between AD probability scores and Aβ status/load. Second, additional parameters could be integrated in the multimodal retinal imaging model to investigate their potential to further improve the discriminatory performance. OCT-angiography [57–60], Doppler OCT [61, 62], and/or systemic variables such as age, sex, APOE status, or blood pressure could provide added value in this respect.

## Conclusion

Retinal imaging offers a fast and straightforward method to examine the central nervous system, allowing direct assessment of neurodegeneration, possibly reflecting Ab deposition or other neurodegenerative-related changes. This study supports the idea that hyperspectral imaging and OCT, combined with a machine learning approach, can contribute to a classification model for the detection of AD. It further supports the idea that this can be achieved with a low-cost, compact, and easy-to-use snapshot camera mounted on top of a standard fundus camera.

## Supplementary Information

**Additional file 1.** Study set-up depicting the multimodal retinal imaging set-up, image processing and analysis.

**Additional file 2.** Details on hyperspectral image analysis.

## Data Availability

The datasets used and/or analyzed during the current study are available from the corresponding author on reasonable request.

## Abbreviations

Aβ: Amyloid-beta
AD: Alzheimer’s disease
ATN: Amyloid-beta, tau, neurodegeneration
AUC: Area under the curve
BCVA: Best corrected visual acuity
CI: Confidence interval
CMOS: Complementary metal oxide semiconductor
CSF: Cerebrospinal fluid
CV: Cross-validation
[^18^F]-FDG PET: [18F] fluorodeoxyglucose PET
IOP: Intraocular pressure
HSRI: Hyperspectral retinal imaging
LDA: Linear discriminant analysis
LOOCV: Leave-one-out cross-validation
MHRC: Metabolic hyperspectral retinal camera
MMSE: Mini-Mental State Examination
OCT: Optical coherence tomography
OD: Optic disc
ONH: Optic nerve head
RNFL: Retinal nerve fiber layer
ROI: Region of interest
SEM: Standard area of the mean
VA: Visual acuity
vCDR: Vertical cup-to-disc ratio
