# Supplementary figures and images for: Combination of snapshot hyperspectral retinal imaging and optical coherence tomography to identify Alzheimer’s disease patients
Source: Alzheimers Res Ther. 2020 Nov 10;12:144. doi: 10.1186/s13195-020-00715-1 (PMC7654576; doi:10.1186/s13195-020-00715-1)

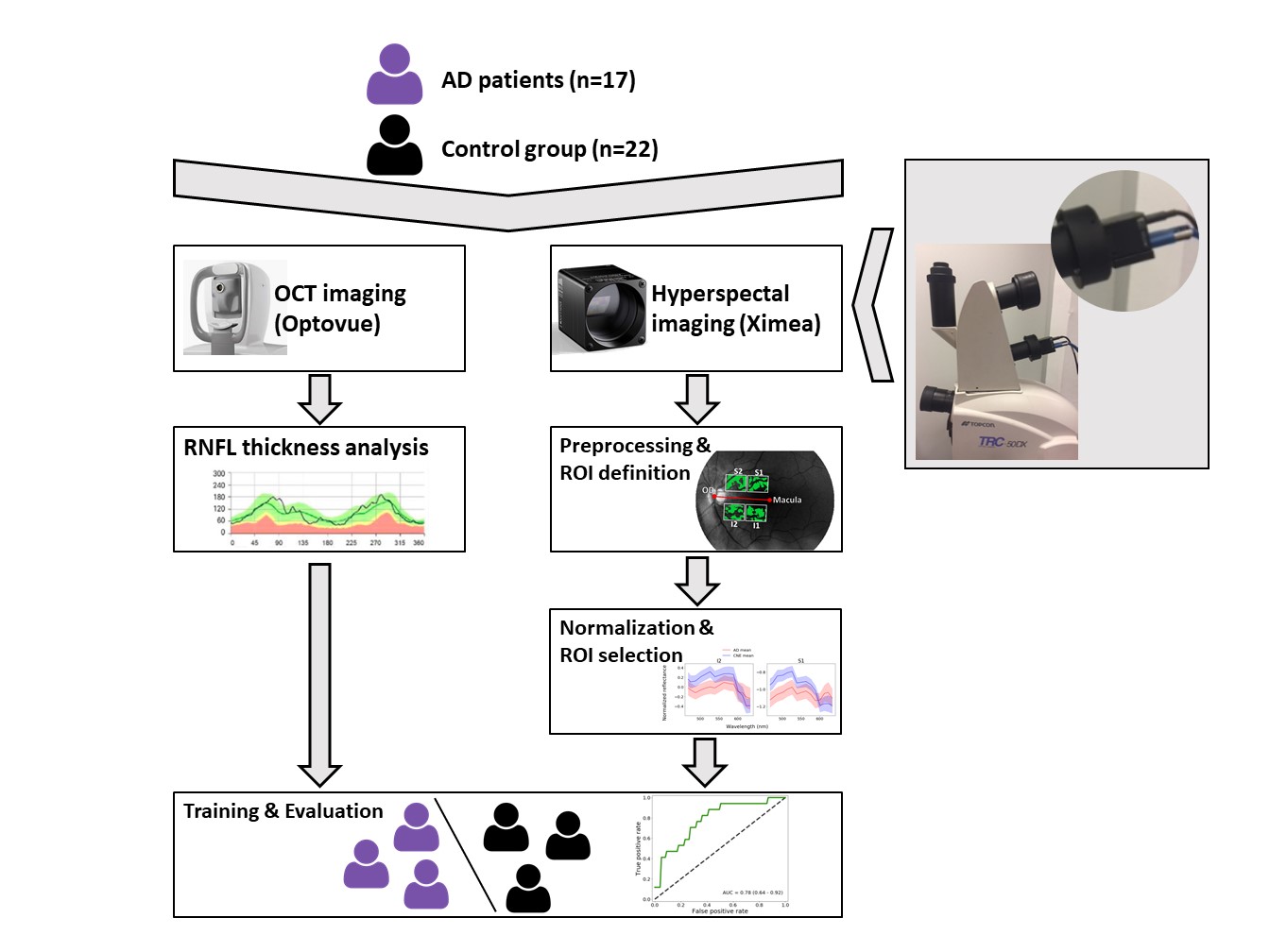

Supplement: Supplementary file 1 — Additional file 1. Study set-up depicting the multimodal retinal imaging set-up, image processing and analysis. [file 13195_2020_715_MOESM1_ESM.jpg]
