# Supplementary material for: Combination of snapshot hyperspectral retinal imaging and optical coherence tomography to identify Alzheimer’s disease patients
Source: Alzheimers Res Ther. 2020 Nov 10;12:144. doi: 10.1186/s13195-020-00715-1 (PMC7654576; doi:10.1186/s13195-020-00715-1)
Supplement: Supplementary file 2 — Additional file 2. Details on hyperspectral image analysis. [file 13195_2020_715_MOESM2_ESM.docx]

**APPENDIX**

Figure A1 presents an overview of the study with the most important steps in data analysis.

**1. Preprocessing**

Relative reflectance (compared to a white reference image) was computed for each hyperspectral image, denoting $R_{i}$ as the reflectance at the $i$-th pixel for a given wavelength:

$$R_{i}=\frac{RS_{i}-RD_{i}}{RW_{i}-R{DW}_{i}}$$

$RS$ refers to the raw intensity values of the sample image, $RD$ refers to the dark reference obtained with the light source turned off and the lens covered, which was used to correct for electrical pixel noise. Furthermore, $RW$ refers to the white reference, obtained by imaging a standard white spectralon tile. *RW* was used to eliminate the spectral profile of the light source. The white reference $RW$ was taken with a shorter exposure time (0.15ms) than $RS$ and $RD$ (0.2ms) to avoid oversaturation. $RDW$ is a second dark reference, taken with an exposure time of 0.15ms to match that of $RW$. Note that no absolute reflectance was calculated as the intensity of the flash of the fundus camera was adapted per patient to maximize the reflected light. Therefore also the (constant) relative ratio of the exposure times of *RS* and *RD* was not used in the calculation of the relative reflectances. Resulting differences in reflectance between subjects were corrected for in the standardization step described below. The resulting relative reflectance images were subsequently multiplied with a spectral correction matrix to correct for cross-talk, leakage, harmonics and camera-specific acquisition artefacts.^1^ These preprocessing steps resulted in a spectrum of relative reflectance values at 14 wavelengths for each hyperspectral pixel (two spectral bands are omitted due to their large signal-to-noise-ratio).

**2. Removal of retinal blood vessels**

Retinal blood vessels were removed from the hyperspectral images by applying a difference of Gaussians (DoG) filter to the entire grayscale image, which was obtained by averaging results over wavelengths 539 nm to 589 nm. This wavelength range was chosen as it most clearly delineates vessel structure, based on visual qualitative assessment. The DoG filter was obtained by applying two Gaussian filters (one with variance 1, the other with variance 10) and subtracting the resulting images. Forty percent of the pixels of the resulting image with the highest intensity were assumed to correspond to vessels and were excluded from further analysis. The 40% cut-off threshold was chosen based on empirical visual inspection of the results of vessel removal, while giving preference to removing background pixels over retaining vessel pixels.

**3. Standardization**

The relative reflectance values of the spectrum were averaged in the four individual ROIs, resulting in a 14x1 vector for each ROI. These spectra were standardized by subtracting the mean spectrum of the entire image, and dividing by the standard deviation of the spectrum of the entire image. This standardization procedure reduces inter-subject variability related to differences in illumination, before performing statistical or machine-learning analysis. The resulting standardized mean spectra were used as input features to the classification model.

**4. Nested leave-one-out cross-validation**

A nested leave-one-out cross-validation (LOOCV) method was used to compare the performance of the four different configurations (2 ROIs that were selected as their SEM does not overlap for at least one wavelength, each input with/without RNFL) and evaluate the resulting classifier (Figure A2). The outer CV procedure was used for model evaluation, whereas the inner CV procedure was used for model selection. In each outer CV iteration, the dataset was split into an outer training dataset consisting of all samples except one and outer test dataset consisting of the remaining sample. An inner CV procedure was then performed on the outer training set to compare the 4 model configurations. The best configuration in this inner CV was selected as the one with the highest AUC computed over the inner CV test set predictions. A model was trained with this configuration on the entire outer training data, and then used to make predictions for the outer test data. This was repeated for all outer CV iterations, and the resulting predictions were used to construct an ROC curve and AUC computation. The 95%-confidence interval for the AUC was computed via the Modified-Wald estimate with continuity correction. Figure A1 illustrates the nested CV procedure with 3-fold CV for illustration purpose. LOOCV was applied in this study on both the outer and inner CV levels.

Figure A2. Illustration of nested cross-validation (CV).


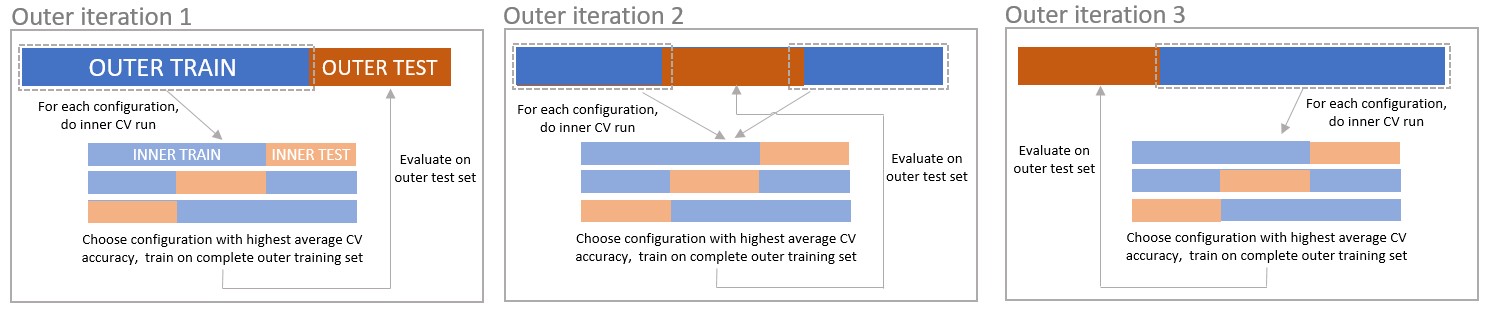


**References:**

1. Pichette J, Goossens T, Vunckx K, Lambrechts A. Hyperspectral calibration method For CMOS-based hyperspectral sensors. In: *Photonic Instrumentation Engineering IV*. ; 2017. doi:10.1117/12.2253617
